# Genetic diversity, variation, and structure of two populations of bigfin reef squid (*Sepioteuthis lessoniana* d’Orbigny) in Con Dao and Phu Quoc islands, Vietnam

**DOI:** 10.1186/s43141-023-00573-y

**Published:** 2023-11-13

**Authors:** Le Ngoc Trieu, Thai Thach Bich, Nguyen Van Ket, Nguyen Van Long

**Affiliations:** 1 The Faculty of Biology, Dalat University (DLU), Lam Dong, Vietnam; 2 Institute of Oceanography, Vietnam Academy of Science and Technology (VAST), Khanh Hoa, Vietnam; 3 Graduated University of Science and Technology (GUST), VAST, Ha Noi, Vietnam

**Keywords:** Bigfin reef squid, SCoT, CBDP, COI, Genetic diversity

## Abstract

**Background:**

Bigfin squid is one of the economically important seafood resources in Vietnam’s fisheries and the waters around Con Dao and Phu Quoc islands are two major fishing grounds where this species has been actively exploited. The start codon targeted polymorphism (SCoT) and CAAT box–derived polymorphism (CBDP) techniques were used to generate DNA fingerprinting data to analyze the genetic diversity, variation, and structure of the two populations in the waters surrounding Phu Quoc and Con Dao islands together with mitochondrial cytochrome C oxidase subunit I (COI) gene sequence data.

**Results:**

Con Dao population possessed a higher diversity [expected heterozygosity (*H*_e_) = 0.2254, Shannon index (*I*) = 0.3459, percentage of polymorphic bands (*PPB*) = 80.14%, nucleotide diversity (*π*) = 0.0336, haplotype diversity (*h*) = 0.910 with 16 haplotypes] than Phu Quoc population (*H*_e_ = 0.1854, *I* = 0.2873, *PPB* = 70.38%, *π* = 0.0246, *h* = 0.838 with 14 haplotypes). The genetic diversity at species level in the investigated region was at level of *H*_e_ = 0.2169, *I* = 0.3399, *PPB* = 86.41, *π* = 0.0289, and *h* = 0.892 with 24 haplotypes. Based on DNA fingerprinting data, the pairwise genetic similarity coefficients among individuals of the Con Dao population were lower (average of 0.7977) than the Phu Quoc population (average of 0.8316). Based on mitochondrial COI data, the pairwise genetic distances among individuals of the Con Dao population were higher (average of 0.0361) than the Phu Quoc population (average of 0.0263). Gene differentiation (*G*_ST_) between two investigated populations was 0.0316 and 0.0310 leading to the genetic distance was 0.0573 and 0.0213 and the gene flow between them was *N*m = 8.2209 and 11.4700 migrants per generation among populations based on DNA fingerprinting and based on COI gene sequence data, respectively. Genetic variation within individuals of both populations (*WP*) played the key role in total genetic variation at species level in surveyed region.

**Conclusions:**

For the bigfin reef squid species in the surveyed region, the Con Dao population had the higher genetic diversity than the Phu Quoc population, between them existed a low to moderate genetic differentiation and a genetic exchange via gene flow. The DNA fingerprinting data better revealed the genetic differentiation between the two surveyed populations while the mitochondrial COI gene sequence data could show the phylogenetic relationship among the surveyed individuals and the other from the sea regions in Southeast Asia. Based on the results obtained, fisheries management strategies are suggested toward the conservation and sustainable exploitation of this species.

**Supplementary Information:**

The online version contains supplementary material available at 10.1186/s43141-023-00573-y.

## Background

The bigfin reef squid, scientifically known as *Sepioteuthis lessoniana* d’Orbigny, is one of the members of the Loliginidae family. This species is considered to be the most widely distributed loliginid squid in the Indo-West Pacific region because besides its largest distribution range from Japan to northern Australia, it also occurs in many other places in the Pacific and Indian Oceans such as New Zealand, the Hawaiian Islands, South India, West Africa, the Madagascar island, Red Sea, and Western Mediterranean [1]. In Vietnam, bigfin reef squid occurs in the waters of the Gulf of Tonkin, the Southern Central, the Southern East, and the Southern West [2–4] and has been considered as one of the most economically important seafood resources.

In its distribution areas, this species usually occurs in water from the surface to a depth of about 100 m, in coastal habitats with the bottom layer usually covered by seagrass beds and coral reefs [1]. Such habitats are relatively common in the Southwestern and Southeastern seas of Vietnam, which are characterized by the coastal habitats of the two large islands, Phu Quoc and Con Dao. In fact, bigfin reef squid used to be fairly abundant and has been considered as a famous seafood in these two islands compared to other distribution areas in Vietnam. These two islands are separated by about 345 km as the crow flies and the shortest sea distance between them is about 410 km. Sharing characteristics with other loliginid squids, the planktonic paralarvae of *S. lessoniana* are widely distributed after hatching by coastal and ocean currents [5, 6] while juveniles and adults live in coastal and consistently move inshore to start mating and spawning [1]. These indicated that adult squid individuals from Con Dao and from Phu Quoc are almost impossible to mate together directly, and therefore bigfin reef squid individuals from Con Dao island and from Phu Quoc island must belong to two separate populations.

Bigfin reef squid in particular and squid in general are crucial components in the food chains and webs as well as the biodiversity of marine ecosystems and are seafood of high economic value for fisheries. Despite playing such important role, the species *S. lessoniana* has faced to two risks, including overexploitation and loss of suitable habitat as a result of climate change [7, 8] which can lead to species extinction and loss of valuable marine resources without effective strategies for conservation and sustainable exploitation.

Such strategies are often established based on a fundamental conception of the importance of intraspecific genetic diversity, which is determined by the genetic diversity of the constituent populations and the gene flow among them and is measured at markers scattered throughout the genome [9]. Low population genetic diversity is often thought to be a consequence of inbreeding degradation and increased genetic drift and this low diversity itself is the cause to reduce individual vitality, along with a depleted capacity for population growth [10]. On the other hand, high genetic diversity is often considered as the driving force to promote population survival and ensure the ability of populations to adapt to changing environmental pressures [11].

In case of the bigfin reef squid in Vietnam, there were several populations which used to be evaluated for genetic diversity, including the population in the Gulf of Tonkin based on the mitochondrial DNA noncoding region [3] and populations in Nha Trang Bay and Phu Quoc island based on mitochondrial COI and 16S rRNA sequences [4]. In the study by Cheng et al. in 2013, the haplotype diversity and the nucleotide diversity were respectively determined at 0.73 and 0.004 for the *S. lessoniana* population in Phu Quoc; however, this data was only based on 17 samples. Thus, population genetic data of bigfin reef squid in Con Dao and Phu Quoc islands have been still lacking and need to be supplemented to create the scientific basis for establishment of conservation and exploitation strategies [4].

For bigfin reef squid, previous studies on population genetic diversity were conducted by two groups of methods, including using mitochondrial DNA sequence data [3, 4] and using DNA fingerprinting data generated through the allozyme marker [12] and microsatellite marker [13, 14].

In general, DNA fingerprinting data can be generated using many markers such as simple sequence repeat (SSR), inter simple sequence repeat (ISSR) [15, 16], random amplified polymorphic DNA (RAPD), and amplified fragment length polymorphism (AFLP) [17]. Although not direct reflecting the allele status of the locus, dominant markers still have been widely used in population genetic diversity assessment due to their high sensitivity in expressing the differences among the surveyed individuals. With the development of molecular biology, many dominant markers have been developed recently, such as start codon targeted polymorphism (SCoT) [18] and CAAT box–derived polymorphism (CBDP) [19] markers. SCoT and CBDP markers target the start codon and CAAT box of functional genes, respectively. Both of these markers are widely used in studies of population genetics in plants; while SCoT markers have also been used in studies of population genetics in animals [20–22], there are almost no found publications on the use of CBDP markers in this field. However, in the gene structure in eukaryotes, the CAAT box is located upstream of the transcription initiation site and signals the binding site for the RNA transcription factor, namely, NF-Y subunits; the sequence of 5′-GGTTA-3′ is conserved for the CAAT box [23].

With the aim of determining the genetic diversity and variation of the two bigfin reef squid populations in Con Dao and Phu Quoc islands and the genetic differentiation between them, in this study, in addition to mitochondrial COI gene sequence data, DNA fingerprinting data generated using two techniques of SCoT and CBDP were also used for analysis.

## Methods

### Materials

Samples were collected in October 2020 by night fishing with lure-hooks in the same way to exploit form local fishermen. In a total of collected 59 juvenile and adult samples of *S. lessoniana*, 28 samples belonged to the Con Dao population were caught in the waters around Con Dao island, and 31 samples belonged to the Phu Quoc population were caught in the waters around Phu Quoc island. Fishing localities of these samples were not more than 10 km offshore. Collected specimens were tentacular tissues of captured individuals, individually labeled and preserved in 95% ethanol at −20 °C until DNA extraction. The field trip sample notation was done continuously for all the caught samples, but only samples belonging to the *S. lessoniana* species were used in this study.

Twenty-eight representative samples of the Con Dao population were assigned as Sles036, Sles037, Sles039–Sles049, Sles052–Sles055, Sles057, Sles058, Sles060–Sles065, Sles067, Sles068, and Sles070. Thirty-one representative samples of the Phu Quoc population were assigned as Sles072–Sles091 and Sles093–Sles103. Samples were not numbered consecutively as above due to exclusion of different species individuals during fishing.

### DNA extraction

Total genomic DNA was extracted from ethanol-preserved tentacular tissue using cetyltrimethylammonium bromide (CTAB) method [24] which modified by Adamkewicz and Harasewych [25]. Three DNA samples were extracted from each individual sample. The DNA concentration and quality were measured using spectrophotometry method [26] using a NanoScan2 system (Analytik Jena). The DNA samples with OD_260_/OD_280_ values between 1.8 and 2.0 were kept at −20 °C for the subsequent PCRs.

### DNA fingerprinting

In this study, DNA fingerprinting data was obtained by using SCoT and CBDP techniques. PCRs were performed in 50 μL reactions containing 25 μL My Red HS Taq mix (Bioline), 0.2 μM primer, and approximately 30 ng DNA templates. The PCRs were performed using an Eppendorf Mastercycler Pro S thermal cycler (Germany). The thermal program used in SCoT technique was as follows: initial denaturation at 94 °C for 5 min; 36 cycles of 94 °C for 15 s, 50 °C for 15 s, 72 °C for 45 s; final extension at 72 °C for 10 min [18]. The thermal program used in CBDP technique: initial denaturation at 94 °C for 5 min; 6 cycles of 94 °C for 45 s, 35 °C for 45 s, 72 °C for 90 s; 30 cycles of 94 °C for 45 s, 51 °C for 45 s, 72 °C for 90 s; final extension at 72 °C for 10 min [19].

After screening, 10 primers for each technique were chosen for PCR; the sequences and amplification features of these primers are shown in Table 1.

**Table 1:** Primers used in the study and their amplification features in total 59 samples of whole species in surveyed region

No	Primer code	Sequence 5′ to 3′	Number of induced bands	Percentage of polymorphic bands (%)
**SCoT technique**				
1	SCoT 6	CAACAATGGCTACCACGC	18	88.89
2	SCoT 9	CAACAATGGCTACCAGCA	15	100.00
3	SCoT 12	ACGACATGGCGACCAACG	28	78.57
4	SCoT 13	ACGACATGGCGACCATCG	22	90.91
5	SCoT 18	ACCATGGCTACCACCGCC	19	89.47
6	SCoT 19	ACCATGGCTACCACCGGC	5	80.00
7	SCoT 22	AACCATGGCTACCACCAC	16	93.75
8	SCoT 29	CCATGGCTACCACCGGCC	2	50.00
9	SCoT 30	CCATGGCTACCACCGGCG	12	91.67
10	SCoT 34	CATGGCTACCACCGGCCC	15	80.00
**CBDP technique**				
11	CBDP 1	TGAGCACGATCCAATAGC	15	100.00
12	CBDP 2	TGAGCACGATCCAATAAT	13	92.31
13	CBDP 3	TGAGCACGATCCAATACC	13	84.62
14	CBDP 4	TGAGCACGATCCAATAAG	11	90.91
15	CBDP 5	TGAGCACGATCCAATCTA	15	93.33
16	CBDP 6	TGAGCACGATCCAATCGA	11	81.82
17	CBDP 7	TGAGCACGATCCAATCGA	13	100.00
18	CBDP 8	TGAGCACGATCCAATCGG	13	84.62
19	CBDP 9	TGAGCACGATCCAATGAT	9	88.89
20	CBDP 10	TGAGCACGATCCAATGTT	14	78.57
	Total		279	
	Average			86.92

To obtain DNA fingerprints for investigated samples, PCR products were separated in 2% agarose gel, electrophoresis was performed at 60 V for 3 h using TBE buffer, the gel then was stained with ethidium bromide (0.5 μg/mL), and photographed under 254/312 nm wavelength lights using UVP GelStudio Plus System (Analytik Jena, Germany).

### COI amplification and sequencing

The DNA sequence of the mitochondrial COI gene was isolated amplified using the primer pairs LCO1490 (5′-GGTCAACAAATCATAAAGATATTGG-3′) and HCO2198 (5′-TAAACTTCAGGGTGACCAAAAAATCA-3′) [27]. PCRs were performed in 60 μL reactions containing 30 μL My Red HS Taq mix (Bioline), 0.4 μM each primer, and approximately 40 ng DNA templates using the Eppendorf Mastercycler Pro S thermal cycler (Germany). The thermal program used to amplify COI gene was as follows: initial denaturation at 95 °C for 5 min; 30 cycles of 95 °C for 15 s, 46 °C for 15 s, 72 °C for 30 s; final extension at 72 °C for 10 min. This program was modified from Folmer et al. [27] and Kim et al. [28] by gradient testing for primer annealing temperature.

PCR products were confirmed by electrophoresis on 1.0% agarose gel electrophoresis and then were purified using AccuPrep® Gel Purification Kits (Bioneer, Korea). Purified DNA samples together with two primers LCO1490 and HCO2198 were preserved in icepack during sending to 1st BASE DNA Sequencing Services (Singapore) for sequencing. DNA sequencing was performed by Sanger method using ABI 3730 XL sequencer.

### Data analysis

The parameters of genetic diversity and variation were calculated for each of two populations and for the whole species in the surveyed region. DNA fingerprint data and mitochondrial COI sequence data were analyzed using different specialized software to calculate the diversity and variation parameters and construct the dendrograms for genetic relationship among studied samples.

#### Based on DNA fingerprinting data

Since both CBDP and SCoT markers are dominant, each observed band was assumed to represent the genotype at a single biallelic locus [29] and DNA fingerprinting data from them can be combined together for genetic analysis (SCoT and CBDP data sets possessed the same overall utility when being applied in samples of whole species in surveyed region, which were analyzed using iMEC—an online marker efficiency calculator, data not shown). The bands were scored as presence (1) or absence (0) characters to construct the binary data matrix. In studies on population genetic diversity and variation, it is generally recognized that increasing the number of investigated loci gives more reliable results [30]. Accordingly, in this study, the analysis was performed with all bands generated using both SCoT and CBDP techniques.

POPGENE 32 software was used to calculate genetic diversity and variation parameters: the percentage of polymorphic bands (*PPB*), the expected heterozygosity (*H*_e_), Shannon index (*I*), the gene differentiation (*G*_ST_), the genetic distance between investigated populations (*D*), and gene flow between them (*N*m) [31]. Pairwise genetic similarity coefficients among investigated samples were calculated and UPGMA dendrograms for the genetic relationship among them were established by using NTSYSpc 2.1 software [32]. Analysis of molecular variance (AMOVA) was carried out using the GenAlEx 6.5 program [33] to describe the distribution of genetic variation among (*AP*) and within (*WP*) investigated populations.

#### Based on the sequence of mitochondrial COI gene

Searching for previously published sequences with high genetic similarity to the samples investigated in the current study using the BLAST tool of the GenBank to show the genetic relationship between the collected samples and representative samples of other distribution regions of the bigfin reef squid. The parameters of nucleotide diversity (*π*), haplotype diversity (*h*), and the distribution of genetic variation among (*AP*) and within (*WP*) investigated populations were calculated using Arlequin software v3.5.1.2 [34]. The number of genotypes in the sample sets, the gene differentiation between the two sample sets (*G*_ST_), and the gene flow between the two sample sets (*N*m) were calculated by the DnaSP software v6.12.01 [35]. The calculation of the pairwise genetic distance between the surveyed samples and the establishment of dendrogram diagrams of the genetic relationship among them were carried out using MEGA7 software [36] using the UPGMA method [37].

## Results

### Characteristics of the obtained data

The results from Table 1 showed that with the same number of used primers, the SCoT technique reflects more loci than the CBDP technique, but the percentage of polymorphic loci under the CBDP technique is higher than that of the SCoT technique.

**Fig. 1:**
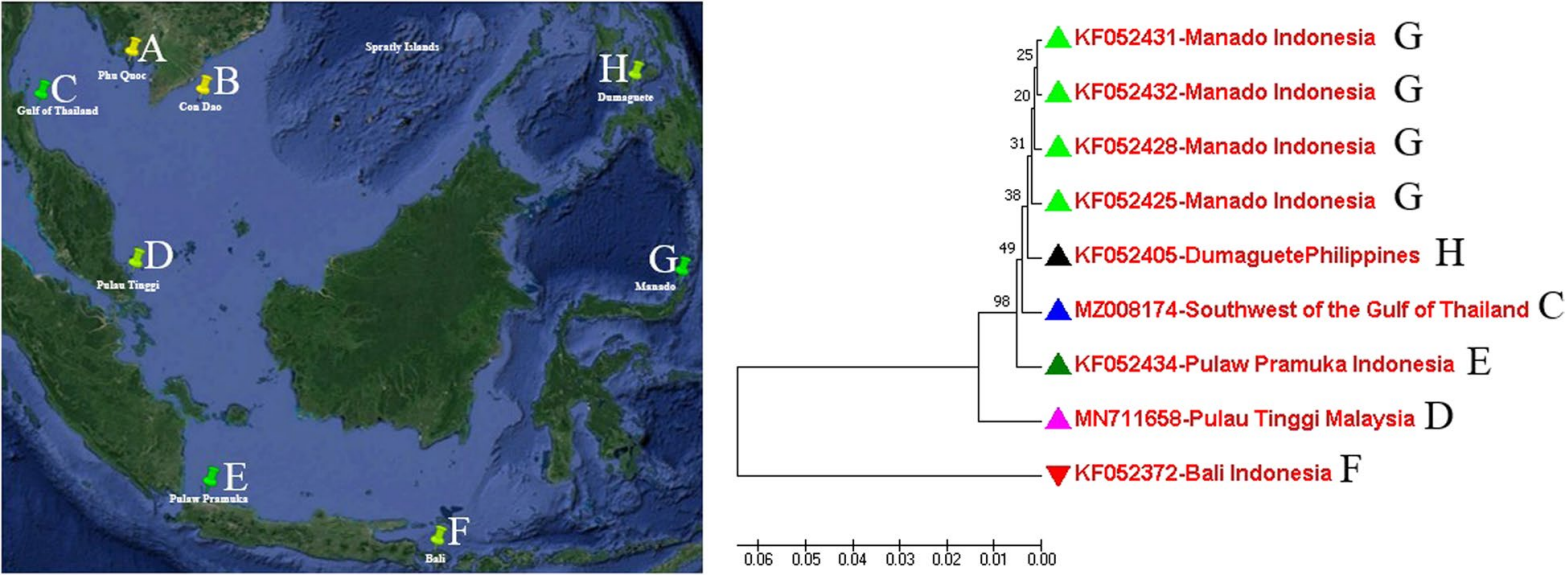
Distribution of bigfin reef squid samples possessed high genetic similarity with the samples collected in current study and the genetic relationship among them. Note: A: Phu Quoc population, B: Con Dao Population. Different color triangles were marked in the dendrogram to indicate the investigated samples in current study which possessed high genetic similarity with the samples from six Southeast Asian waters in following COI-based dendrograms

After sequencing, the corresponding COI sequence region between the investigated samples was 543 bp in length; this is the partial sequence of the COI gene used in further analysis. These sequences had high genetic similarity (>99.6%) with the corresponding sequences recorded for bigfin reef squid in 6 areas in Southeast Asian waters that have been published in GenBank. These areas include southwest of the Gulf of Thailand (C); Pulau Tinggi, Malaysia (D); Pulaw Pramuka, Indonesia (E); Bali, Indonesia (F); Manado, Indonesia (G); and Dumaguete City, Philippines (H) which are indicated in Fig. 1 together with the dendrogram for the genetic relationship among the representative samples.

The detail of DNA fingerprinting data and COI sequence data for investigated samples was shown in Supplementary data.

### Genetic diversity at population and species levels in investigated region

The genetic diversity parameters of the two surveyed populations are shown in Table 2.

**Table 2:** Genetic diversity of Con Dao and Phu Quoc populations

	Parameters	Con Dao population	Phu Quoc population
Based on DNA fingerprinting data	Expected heterozygosity (*H*_e_)	0.2254	0.1854
	Shannon index (*I*)	0.3459	0.2873
	Percentage of polymorphic bands (*PPB*)	80.14%	70.38%
Based on DNA sequence of the COI gene	Nucleotide diversity (*π*)	0.0336	0.0246
	Haplotype diversity (*h*)	0.910	0.838
	Number of genotypes	16	14

All genetic diversity parameters based on DNA fingerprinting data and based on mitochondrial COI sequences in Table 2 showed that the Con Dao population had a higher genetic diversity than the Phu Quoc population.

At the species level in the investigated region included 59 individuals, genetic diversity parameters based on combined DNA fingerprinting data were *H*_e_ = 0.2169, *I* = 0.3399, and *PPB* = 86.41; based on mitochondrial COI gene sequence data, the diversity parameters were *π* = 0.0289 and *h* = 0.892 with 24 genotypes.

### Genetic structure of the two populations and whole species in investigated region

#### Con Dao population

**Fig. 2:**
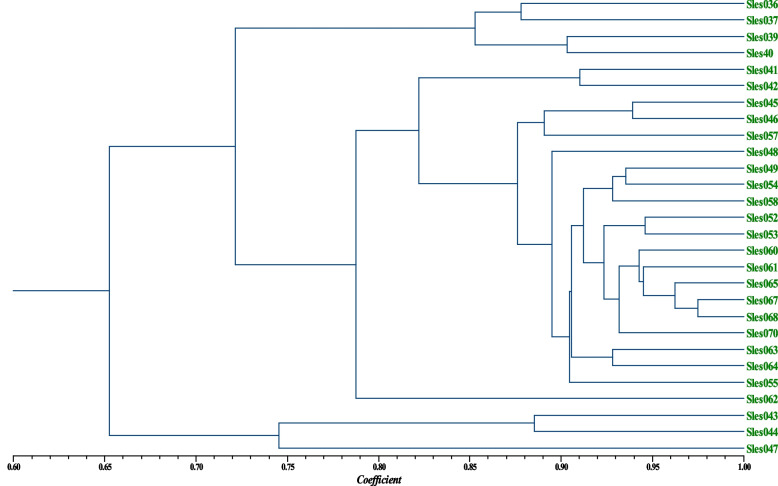
UPGMA dendrogram for the genetic relationship among individuals of Con Dao population based on DNA fingerprinting data

The pairwise genetic similarity coefficients among individuals of the Con Dao population based on DNA fingerprinting data were in range of 0.5484–0.9749 with an average of 0.7977. Based on these genetic similarity coefficients, the dendrogram for genetic relationship among individuals was established and shown in Fig. 2.

In Fig. 2, it is clearly to recognize that the three samples of Sles043, Sles044, and Sles047 formed a separate group from the rest of the population.

**Fig. 3:**
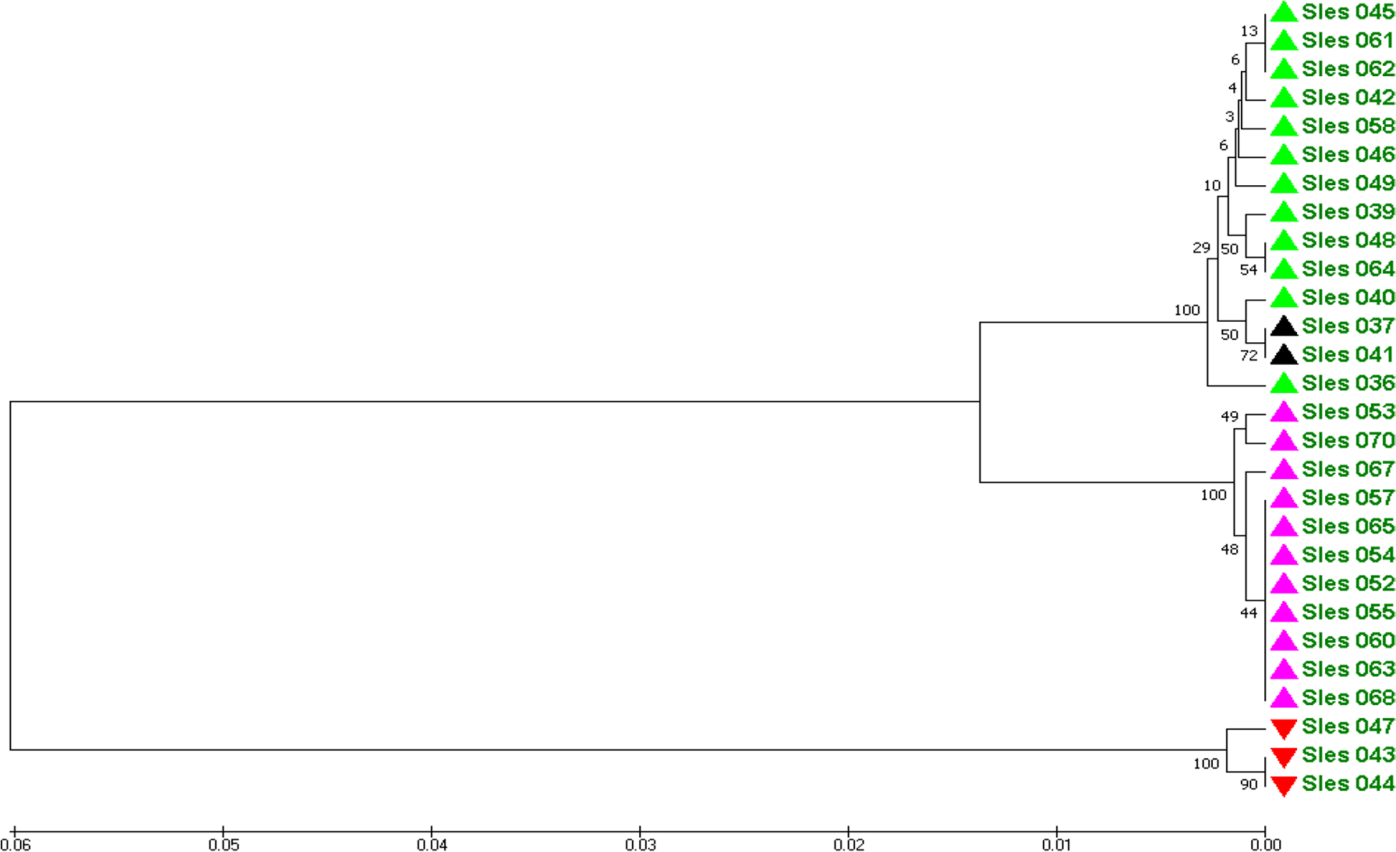
The UPGMA dendrogram for the genetic relationship among individuals of Con Dao population based on mitochondrial COI data

Based on mitochondrial COI data, the genetic distances among samples of the Con Dao population ranged from 0.000 to 0.1315 with a mean value of 0.0361. The UPGMA dendrogram was constructed based on these genetic distances and is shown in Fig. 3.

In Fig. 3, three samples of Sles043, Sles044, and Sles047, which have high genetic similarity with the sample from Bali, Indonesia, also formed a separate group from the rest of the population.

#### Phu Quoc population

**Fig. 4:**
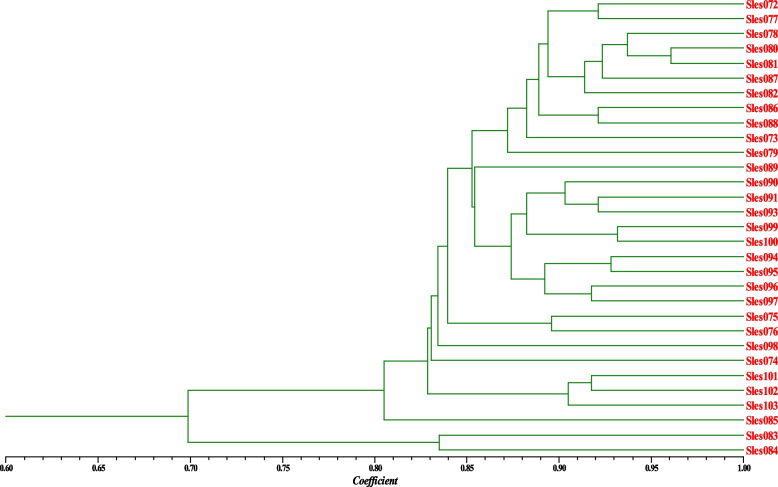
UPGMA dendrogram for the genetic relationship among individuals of Phu Quoc population based on DNA fingerprinting data

The pairwise genetic similarity coefficients among individuals of the Phu Quoc population based on DNA fingerprinting data were in range of 0.5950–0.9606 with an average of 0.8316. Based on these genetic similarity coefficients, the dendrogram for genetic relationship among individuals was established and shown in Fig. 4.

In Fig. 4, it is easy to recognize that the two samples of Sles083 and Sles084 formed a separate group from the rest of the population.

**Fig. 5:**
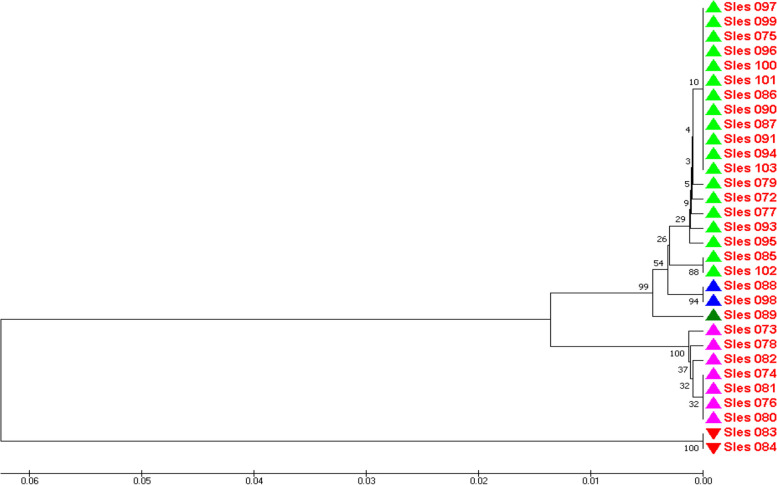
The UPGMA dendrogram for the genetic relationship among individuals of Phu Quoc population based on mitochondrial COI data

Based on mitochondrial COI data, the genetic distances among samples of the Phu Quoc population ranged from 0.000 to 0.1315 with a mean value of 0.0263. The UPGMA dendrogram was constructed based on these genetic distances and is shown in Fig. 5.

From Fig. 5, it can be seen that two samples of Sles083 and Sles084, which have high genetic similarity with the sample from Bali, Indonesia, also form a separate group from the rest of the population.

The similarity between dendrograms based on DNA fingerprinting data and based on COI sequence data was that both reflected the separation of samples Sles043, Sles044, and Sles047 from Con Dao population and of samples Sles083 and Sles084 from Phu Quoc population.

#### Whole species in investigated region

**Fig. 6:**
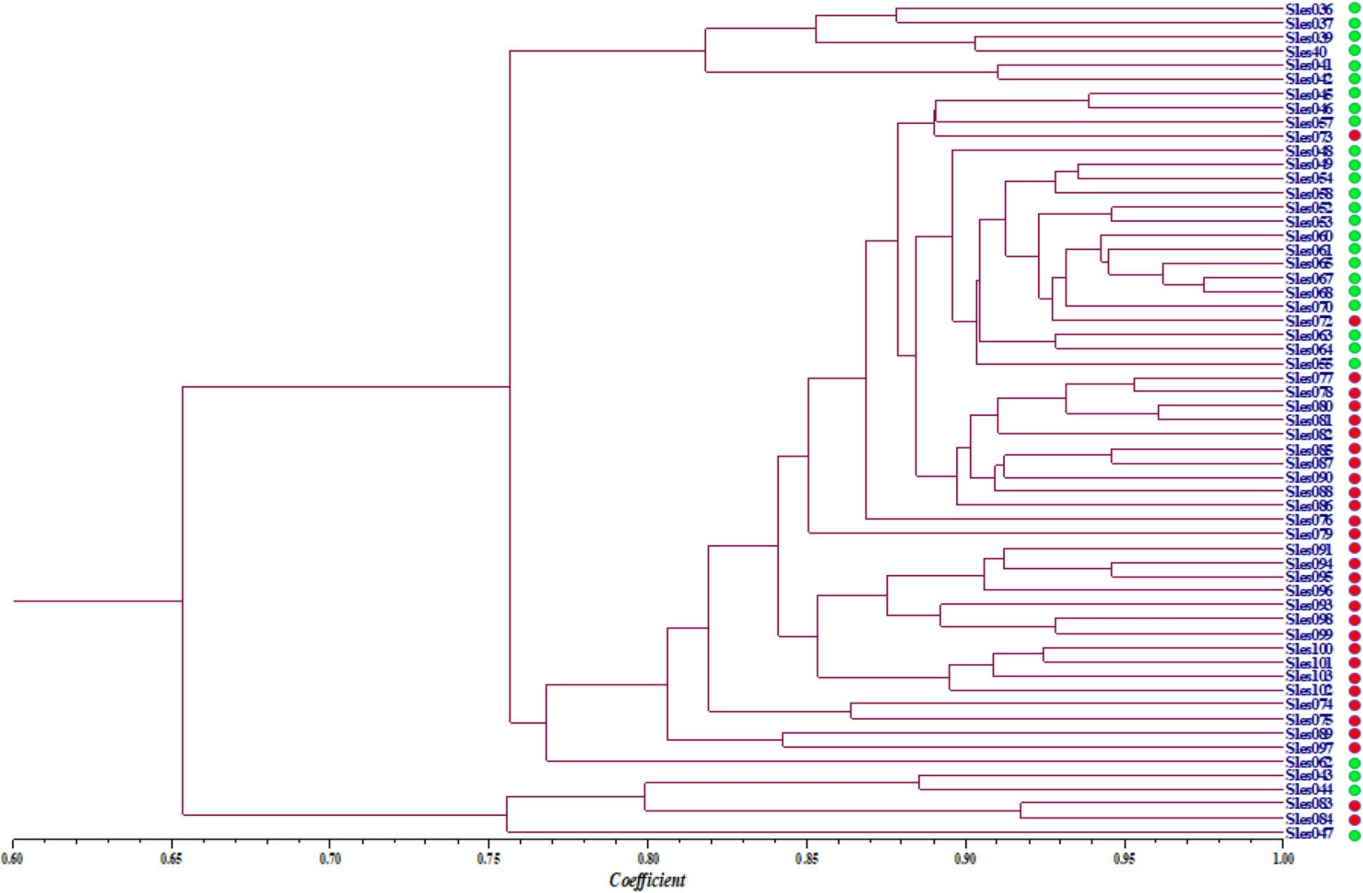
UPGMA dendrogram for the genetic relationship among 59 individuals of whole species in investigated region based on DNA fingerprinting data

The pairwise genetic similarity coefficients among 59 individuals of both Con Dao and Phu Quoc populations based on DNA fingerprinting data were in range of 0.5484–0.9749 with an average of 0.8073. Based on these genetic similarity coefficients, the dendrogram for genetic relationship among individuals was established and shown in Fig. 6.

In Fig. 6, the five samples of Sles043, Sles044, Sles047, Sles083, and Sles084 formed a separate group. For the rest 54 samples, most of the samples from the same population grouped closely together, except two samples of Sles073 and Sles073 from the Phu Quoc population that grouped with the samples of the Con Dao population and the sample Sles062 of the Con Dao population that self-formed a separate group.

**Fig. 7:**
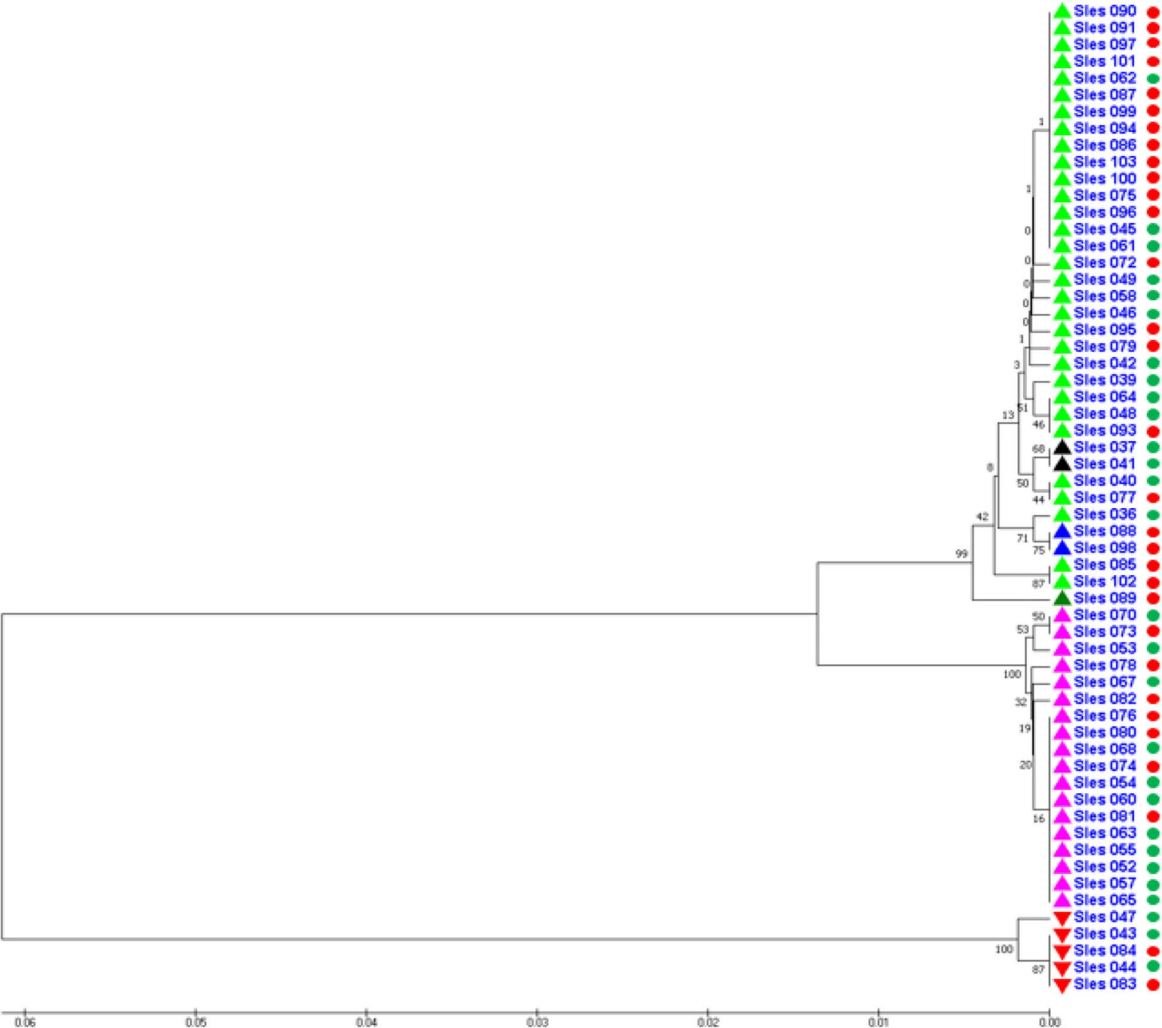
UPGMA dendrogram for the genetic relationship among 59 individuals of whole species in investigated region based on mitochondrial COI data

Based on mitochondrial COI data, the genetic distances among samples of 59 individuals of both Con Dao and Phu Quoc populations ranged from 0.000 to 0.1315 with an average value of 0.0311. The UPGMA dendrogram was constructed based on these genetic distances and is shown in Fig. 7.

In Fig. 7, five samples of Sles043, Sles044, Sles047, Sles083, and Sles084, which possess high genetic similarity with the sample from Bali, Indonesia, also form a separate group from the rest of the total. There was the interlaced clustering of samples from both surveyed populations. The samples with high genetic similarity to samples from each of different sea waters in Southeast Asia clustered closely together.

Parameters of the gene differentiation, genetic distance between investigated populations, gene flow between them, and distribution of genetic variation in whole species in surveyed region are indicated in Table 3.

**Table 3:** The gene differentiation, genetic distance between investigated populations, gene flow between them, and distribution of genetic variation at species level in surveyed region

Parameters	Based on DNA fingerprinting data	Based on COI sequence
Genetic distance between investigated populations (*D*)	0.0316	0.0310
Gene differentiation (*G*_ST_)	0.0573	0.0213
Gene flow between investigated populations (*N*m)	8.2209	11.4700
Distribution of genetic variation among investigated populations (*AP*)	7.42%	0.88%
Distribution of genetic variation within individuals from both investigated populations (*WP*)	92.58%	99.12%

The results from Table 3 showed the existence of genetic differentiation and also genetic exchange between the Con Dao population and the Phu Quoc population.

In Figs. 6 and 7, the individuals from Con Dao population were marked as the red circles and the individuals from Phu Quoc population were marked as the green circles.

## Discussion

The higher genetic diversity of the Con Dao population comparing to Phu Quoc population can be explained by the planktonic paralarvae dispersal behavior of the species and by coastal and ocean currents in the studied region. During the northeast monsoon season (January to March), there has been a strong current from through the Luzon Strait to Hainan island and along the coast of Vietnam through Con Dao island, Malaysia to the Karimata Strait and another weak current from Con Dao island to Phu Quoc island. During the southwest monsoon season (July to September), there has been a strong current from the Java Sea through the Karimata Strait, Malaysia, the mouth of the Gulf of Thailand to Con Dao Island and then has flowed toward the northeast and another weak current from Phu Quoc island to Con Dao island [38]. With such currents, the Con Dao population has a much higher ability to receive the planktonic paralarvae dispersed from other populations in Southeast Asian waters than the Phu Quoc population.

Population genetic diversity in bigfin reef squid has been reported in previous studies. Using the allozyme technique, Pratoomchat et al. showed that the observed heterozygosity and *PPB* of the populations at Nagasaki (Japan) and at Rayon (Thailand) were 0.28; 45.45% and 0.23; 50%, respectively [12]. Using the SSR technique, Tomano et al. showed that the observed heterozygosity of the population in Mugi (Japan) was 0.697 and Zheng et al. indicated the expected heterozygosity of the population in Hainan island was 0.852 [13, 14]. Compared with these results, the genetic diversity levels based on DNA fingerprinting data using SCoT and CBDP techniques of the Con Dao and Phu Quoc populations were relatively low.

Based on the mitochondrial DNA non-coding region, Aoki et al. showed that the nucleotide diversity and haplotype diversity of bigfin reef squid populations in Japan, Taiwan, and Gulf of Tonkin of Vietnam ranged from 0.0003 to 0.0124 and from 0.0667 to 0.8972, respectively [3]. Using mitochondrial COI sequence to assess genetic diversity of 13 populations of bigfin reef squid in Southeast Asia, Cheng et al. showed the nucleotide diversity in the range 0.001 to 0.014, with average of 0.005 and the haplotype diversity in the range from 0.59 to 0.95 with average of 0.81. Compared with these results, the genetic diversity of Con Dao and Phu Quoc populations based on COI sequence in current study were at relatively high level [4].

The results about the grouping of the individuals in each of populations and whole species in investigated region suggested that the DNA fingerprinting data better reflects the genetic differentiation between the two surveyed populations comparing to COI sequence data due to individuals from the same population grouped together more closely. The mitochondrial COI sequence data reflects the phylogenetic relationships of the surveyed individuals to the bigfin reef squid individuals in different sea waters of Southeast Asia due to individuals from both two populations were interlaced when grouping and individuals grouped together according to the tendency that individuals possess high genetic similarity to corresponding sequence recorded for *S. lessoniana* at each sea water grouped together closely.

The genetic relationship dendrograms based on DNA fingerprinting data and on mitochondrial COI sequence data shared the same feature that was the separation of the samples of Sles043, Sles044, Sles047, Sles083, and Sles084 from the rest of the population or of whole species in surveyed region. This suggested that these samples were taken from migrants or were the descendants of migrants that may have migrated across large geographic barriers from other populations. In other words, these samples were the consequence of relatively recent gene flows; this was significantly in agreement with their COI gene sequences, which were highly homologous [99.63% (Sles047) and 100% (Sles043, Sles044, Sles083, and Sles084)] to the corresponding sequence recorded for bigfin reef squid in Bali, Indonesia (GenBank accession number of KF052372).

The gene flow between the two surveyed populations could be supported by the dispersal of planktonic paralarvae by two weak currents connecting the Con Dao and Phu Quoc sea waters in the two monsoon seasons [38].

DNA fingerprinting data, which showed close grouping of individuals by population, again better reflects the genetic differentiation and the genetic variation between the two populations compared to mitochondrial COI sequence data. This could be explained by the fact that the SCoT and CBDP data reflect the genetic variation of many genes across different regions of the genome while the mitochondrial COI sequence data only reflect the genetic variation of a single gene.

Analysis of molecular variance (AMOVA) revealed high genetic variation within populations and low genetic differentiation among two populations in investigated regions. Based on the mitochondrial DNA non-coding region, Aoki et al. showed that pairwise gene differentiations among 7 bigfin reef squid populations in Japan, Taiwan, and Gulf of Tonkin of Vietnam were in range of 0.0041 to 0.8593 with the average of 0.3695 [3]. Comparing to this study and the interpretation of gene differentiation values in previous studies [39, 40], the gene differentiation between Con Dao and Phu Quoc populations based on DNA fingerprinting data was at moderate level and based on mitochondrial COI sequence data was at relatively low level.

Based on DNA fingerprinting data, the genetic distance between Con Dao and Phu Quoc populations in current study (*D* = 0.0361) was significantly higher than between Nagasaki and Rayon populations (*D* = 0.003, using allozyme technique) [12], although the geographical distance between the Nagasaki and Rayon populations is much farther than between the Con Dao and Phu Quoc populations.

Using mitochondrial COI sequence to investigate 11 populations of bigfin reef squid in Southeast Asia, Cheng et al. showed that the significantly different genetic variation distribution among populations (77.51% or 23.24%) and within populations (22.49% or 76.76%) depended on the investigate lineages [4]. Compared with the results from this study, genetic variation within populations in current study was much higher. This may due to in the presentative sample sets for Con Dao and Phu Quoc populations included different lineages of bigfin reef squid which inherently identified by Cheng et al. and the surveyed region in current study was also smaller.

As mentioned above, the information on the genetic diversity, differentiation, and gene flow among the populations is necessary to establish the strategies for conservation and sustainable exploitation of the species. The findings from the current study indicate that the Con Dao population had a higher genetic diversity than the Phu Quoc population. The main reason for that difference may be that Con Dao island is exposed to strong ocean currents from two different directions, while there has been only a weak ocean current bringing planktonic paralarvae to Phu Quoc island. Accordingly, to maintain the long-term genetic diversity of the populations in surveyed regions, several fishery management strategies including the promotion of programs of artificial propagation and local as well as translocational releasing to increase the size of local populations and to overcome barriers to gene flows among populations; establishing regulations to limit fishing in the breeding season; and enhancing the protection and restoration of coral reefs and seagrass beds to ensure the quality of such habitats, especially at spawning grounds for the species should be fully considered.

## Conclusions

For the bigfin reef squid species in the surveyed region, the Con Dao population had the higher genetic diversity than the Phu Quoc population; however, the levels of genetic diversity based on COI data of these populations were at relatively higher compared to other populations in Southeast Asia. Between the investigated populations existed a low to moderate genetic differentiation and a genetic exchange via gene flow.

DNA fingerprinting data better showed the gene differentiation between the two investigated populations than the mitochondrial COI gene sequence data; the COI gene sequence data reflects the phylogenetic relationships of the individuals investigated in the current study with bigfin reef squid in some other sea areas in Southeast Asia.

From this study, several fishery management strategies for conservation and sustainable exploitation of bigfin reef squid are proposed.

### Supplementary Information

**Additional file 1.**

## Data Availability

The datasets supporting the conclusions of this article are included within the article and its supplementary information files.

## Abbreviations

AMOVA: Analysis of molecular variance
*AP*: Distribution of genetic variation among populations
CBDP: CAAT box–derived polymorphism
COI: Cytochrome C oxidase subunit 1
CTAB: Cetyltrimethylammonium bromide
*D*: Genetic distance between investigated populations
*G*_ST_: Gene differentiation
*h*: Haplotype diversity
*H*_e_: Expected heterozygosity
*I*: Shannon index
NF-Y: Nuclear factor Y
*N*m: Gene flow
OD_260_: Optical density at 260 nm
OD_280_: Optical density at 280 nm
PCR: Polymerase chain reaction
*PPB*: Percentage of polymorphic bands
SCoT: Start codon targeted polymorphism
TBE: Tris-borate-EDTA
UPGMA: Unweighted pair group method with arithmetic mean
*WP*: Distribution of genetic variation within populations
*π*: Nucleotide diversity
